# Differential Role of Sex and Age in the Synaptic Transmission of Degus (*Octodon degus*)

**DOI:** 10.3389/fnint.2022.799147

**Published:** 2022-02-28

**Authors:** Carolina A. Oliva, Daniela S. Rivera, Trinidad A. Mariqueo, Francisco Bozinovic, Nibaldo C. Inestrosa

**Affiliations:** ^1^Center of Aging and Regeneration UC, Departamento de Biología Celular y Molecular, Facultad de Ciencias Biológicas, Pontificia Universidad Católica de Chile, Santiago, Chile; ^2^GEMA Center for Genomics, Ecology & Environment, Facultad de Estudios Interdisciplinarios, Universidad Mayor, Santiago, Chile; ^3^Centro de Investigaciones Médicas, Laboratorio de Neurofarmacología, Escuela de Medicina, Universidad de Talca, Talca, Chile; ^4^Center of Applied Ecology and Sustainability, Departamento de Ecología, Facultad de Ciencias Biológicas, Pontificia Universidad Católica de Chile, Santiago, Chile; ^5^Centro de Excelencia en Biomedicina de Magallanes, Universidad de Magallanes, Punta Arenas, Chile

**Keywords:** *Octodon degus*, synaptic plasticity, basal synaptic transmission, aging, sex differences

## Abstract

*Octodon degus* are a diurnal long-lived social animal widely used to perform longitudinal studies and complex cognitive tasks to test for physiological conditions with similitude in human behavior. They show a complex social organization feasible to be studied under different conditions and ages. Several aspects in degus physiology demonstrated that these animals are susceptible to environmental conditions, such as stress, fear, feeding quality, and isolation. However, the relevance of these factors in life of this animal depends on sex and age. Despite its significance, there are few studies with the intent to characterize neurological parameters that include these two parameters. To determine the basal neurophysiological status, we analyzed basic electrophysiological parameters generated during basal activity or synaptic plasticity in the brain slices of young and aged female and male degus. We studied the hippocampal circuit of animals kept in social ambient in captivity under controlled conditions. The study of basal synaptic activity in young animals (12–24 months old) was similar between sexes, but female degus showed more efficient synaptic transmission than male degus. We found the opposite in aged animals (60–84 months old), where male degus had a more efficient basal transmission and facilitation index than female degus. Furthermore, female and male degus develop significant but not different long-term synaptic plasticity (LTP). However, aged female degus need to recruit twice as many axons to evoke the same postsynaptic activity as male degus and four times more when compared to young female degus. These data suggest that, unlike male degus, the neural status of aged female degus change, showing less number or functional axons available at advanced ages. Our data represent the first approach to incorporate the effect of sex along with age progression in basal neural status.

## Introduction

Among animal models, the endemic Chilean rodent degus (*Octodon degus*) convey unique features considered relevant as a model intended to extrapolate human characteristics. This species lives in a complex organized society where they develop solid social behaviors that include social-affective bonds. In addition, they are extremely sensitive to external conditions throughout their life (Fulk, 1976; Colonnello et al., 2011). Degus are diurnal and long-lived animals used in several studies, including the analysis of numerous social conditions (Helmeke et al., 2009; Pereda-Pérez et al., 2013; Quispe et al., 2014; Rivera et al., 2020, 2021a) or as a model for different diseases or treatments (Ardiles et al., 2012; Rivera et al., 2016; Cisternas et al., 2018), due to its facility to examine high-order cognitive behaviors through different tools (Popović et al., 2010; Tarragon et al., 2013, 2014). The study of the neurological aspects involved in cognitive and social behavior and how they evolve during disease or environmental conditions must consider the living context, age, and sex of animals.

Aging has become a central topic since the expectation of human life increased. Changes in the behavioral, physiological, and molecular aspects of age are under extensive scrutiny in animal models and humans. Recently, degus were considered an appropriate model due to extraordinary similarities to the aging process in humans (Inestrosa et al., 2005; Cisternas et al., 2018). Like humans, aged degus show detrimental physical, physiological, and cognitive performance, reducing their capacity to react to environmental conditions with an apparent reduction in several cognitive capacities and memory performance, increasing the possibility to develop pathologies. Indeed, aged degus naturally develop features similar to Alzheimer’s disease (AD) (Cisternas et al., 2018; Hurley et al., 2018). At over 36 months old, degus show diminished synaptic activity, and defects on some postsynaptic mechanisms appear to be the main cause (Ardiles et al., 2012; Rivera et al., 2016). Accumulation of Aβ oligomers, amyloid plaques, and tau hyperphosphorylation are significant in aged degus and are responsible for cognitive disturbances and death. Notably, aged degus can recover abilities that involve recognition memory, improve synaptic strength, and synaptic function (Rivera et al., 2016), demonstrating that it is also a model to test treatments. Nevertheless, most studies in rodents and degus do not distinguish between sexes assuming that standard aging features affect both sexes equally.

The study of sex differences is bypassed most of the time. The use of male degus has been justified to avoid hormonal estrous cycling to reduce experimental variability (Beery, 2018). The argument that a biological relevant mechanism should be sex-independent is often used to focus on the broad aspects of similarities rather than on differences. However, specific differences may hideout the solution for specific treatments. The evidence has pointed out that species with complex and evolved social colony system like degus showed significant biological differences. For example, Popović et al. (2010) showed sex differences in spatial learning and memory. Moreover, recent studies evaluating the impact of isolation at different ages in female and male degus (Rivera et al., 2020, 2021a) show that under different stress levels in life, degus display a reduction in social motivation, synaptic transmission, and increased occurrences of impaired cognition (Colonnello et al., 2011; Rivera et al., 2020). Synaptic proteins, synaptic plasticity paradigms (long-term plasticity, LTP), and spatial memory or social performance were quantifiable measurements obtained from these studies, with measurable differences between sexes (Rivera et al., 2020, 2021a). Social isolation stress affected the cognitive performance of male degus, not female degus, while the affective and social memory is more affected in female degus. Like degus, humans, in some degree of social deprivation, manifest the risk of psychopathy, which can affect individuals differently depending on age and sex (Dekker et al., 2007; Zahn-Waxler et al., 2008). This is in terms of pathological conditions, but gender differences have also been reported on normal conditions and in performing regular activities (Hirnstein et al., 2019; Jockwitz et al., 2021).

Whether these differences in sex and age are manifested in anomalous or exacerbated conditions or are part of basal differences undetected before is a matter of study. To determine this last point, we explore the parameters that underlie the basal synaptic transmission and the display of synaptic plasticity. We used young and aged female and male degus to study the electrophysiological properties of neuronal activity relevant during basal and LTP at two age ranges. We found intrinsic differences between female and male degus, exacerbated during aging, suggesting that some components or mechanisms related to sex differentiation were previously underestimated in their neurological effect.

## Materials and Methods

### Animals

Female and male degus of different ages (12–84 months old) derived from laboratory-bred lines were obtained from our colony at the Faculty of Biological Sciences, Pontificia Universidad Católica de Chile. Degus were kept in sex-matched pairs of related and unrelated brothers or sisters and housed in clear acrylic terrariums (length × height × depth: 50 cm × 35 cm × 23 cm) with bedding of hardwood chips. Water and food (commercial rabbit pellet; Champion, Santiago, Chile) were provided *ad libitum*. Each cage contained 1 nest box made of clear acrylic (22 cm × 12 cm × 15 cm). Animals were kept in a ventilated, temperature-controlled (yearly minimum = 13.4 ± 0.2°C; yearly maximum = 24.9 ± 0.2°C) room exposed to a 12:12 h light: dark cycle. All animal protocols followed the National Institutes of Health guide (NIH, Baltimore, MD, United States; NIH Publications No. 8023, revised 1978) for the care and use of laboratory animals. The Bioethical and Biosafety Committee approved all procedures of the Faculty of Biological Sciences of the Pontificia Universidad Católica de Chile (CBB-121-2013). Efforts were made to minimize animal suffering and reduce the number of animals used.

### Electrophysiology

The procedures for slice preparation were written in detail by Rivera et al. (2020). Animals were separated into two groups: young female and male degus (12–24 months old) and aged female and male degus (60–84 months old). Briefly, degus were euthanized by decapitation after anesthesia. The brains were quickly removed and placed in modified cold artificial cerebrospinal artificial solutions (ACSFs; sucrose replaces part of sodium) composed of the following (in mM): 85 NaCl, 75 sucrose, 3 KCl, 1.25 NaH_2_PO_4_, 25 NaHCO_3_, 10 dextrose, 3.5 MgSO_4_, 0.5 CaCl_2_, 3 sodium pyruvate, 0.5 sodium L-ascorbate, and 3 Myo-inositol (305 mOsm, pH 7.4), before being oxygenated (95% O_2_/5% CO_2_). We cut coronal section slices (300–350 μm) with a vibratome, and the slices were allowed to recover for an hour in the same solution. After that, we changed the solution to an oxygenated ‘recording solution’ composed of (in mM): 126 NaCl, 3.5 KCl, 1.25 NaH_2_PO_4_, 25 NaHCO_3_, 10 dextrose, 1 MgSO_4_, 2 CaCl_2_, 3 sodium pyruvate, 0.5 sodium L-ascorbate, and 3 Myo-inositol (305 mOsm, pH 7.4) at room temperature (22°C) where slices were kept until recording, during which the temperature was raised to 35–36°C. For recording, each slice was placed under an upright infrared-differential interference contrast (IR-DIC) fluorescence microscope (Eclipse FNI, Nikon), the hippocampal circuit visualized with a 40x water objective, and the Schaffer collaterals between CA3 and CA1 and stimulated using a bipolar concentric electrode (World Precision Instruments, Sarasota, FL, United States) connected to an ISO-Flex stimulus generator (AMPI, Jerusalem, Israel). We used a glass electrode (World Precision Instruments, Sarasota, FL, United States) of 0.5–1 MΩ pulled on a P-97 Micropipette Puller (Sutter Instruments, Novato, CA, United States) filled with recording solution. The electrode was placed on the *stratum radiatum* of CA1 to record the evoked field excitatory postsynaptic potentials (fEPSPs). The signals were recorded using a MultiClamp 700B amplifier (Axon CNS, Molecular Devices LLC, San Jose, CA, United States) and digitally sampled at 30 kHz using a Digidata-1440A interface (Axon CNS, Molecular Devices LLC, San Jose, CA, United States). The analyses were performed offline using pClamp 10.3 (Molecular Devices LLC, San Jose, CA, United States). We applied several protocols to characterize the basal status of this synapse: (i) The input-output curve, where increasing levels of current intensity were successively applied, and we plotted the fEPSP slopes and fiber volley (FV) amplitudes obtained. The amount of current to evoke 60% of the maximum slope value was used to perform other protocols in the same slice. (ii) Paired-pulse facilitation (PPF) protocol was induced by applying two pulses separated by different times (inter-stimulus interval, ISI), and the R2/R1 ratio obtained was used to estimate the degree of facilitation. (iii) Synaptic plasticity was induced using LTP protocols. Two pulses (R1 and R2, separated by 50 ms each) were evoked every 15 s. The fEPSP slope of the first pulse (R1) was averaged for 15–20 min to obtain a stable basal signal. Theta-burst stimulation (TBS, 5 bursts at 100 Hz every 20 s) was applied to induce LTP. The post-TBS was evaluated using the identical two pulses separated by 50 ms for at least 60 min.

### Statistical Analysis

For this study, we used *n* = 7 for young female degus, *n* = 5 for aged female degus, *n* = 9 for young male degus, and *n* = 7 for aged male degus. At least three slices from each animal were considered replicates, averaged together, and counted like *n* = 1. Data are expressed as mean ± SEM. Electrophysiological data to measure Input–Output curves were analyzed *via* two-way ANOVA to determine the effect of sex and stimulus intensity, or age and stimulus intensity, and the interaction between the two factors. To determine the differences in synaptic transmission efficacy, we plotted the FV amplitude value and its corresponding fEPSP slope. The points were adjusted with linear regression. To further compare whether the regression lines are different, we used the analysis of covariance (ANCOVA). This analysis tests the null hypothesis that both regressions are identical in slope and intercept. We applied this analysis in our data to determine the difference between our experimental groups. The PPF protocol was analyzed with two-way ANOVA to determine the effect of sex and inter-stimulus interval, or age and inter-stimulus interval, and the interaction between the two factors. We analyzed the LTP experiments by comparing the average of the last 10 min of recordings between young and aged female and male degus using one-way ANOVA. In all cases, the Bonferroni *post hoc* test was used. Using Pearson’s correlation, we found the correlation between FV amplitude and fEPSP slope before or after TBS. Using Fisher ‘*r*’ to ‘*z*’ transformation, we used *z*-scores to compute the significance of the difference when we compared two correlation coefficients. For the attenuation plot, we plotted the R1 (%) and R2 (%) and used the formula (R2-R1)/R1 (%). The two-way ANOVA determined the differences between time course and sex, or time course and age, and the interaction between both factors. Additionally, one-way ANOVA was performed over the average of the last 10 min recordings to assay differences within all groups. For TBS analysis, we plotted the first pulse of each burst (from 1st to 5th), normalized to the first (1st). For the area calculation, each burst excitatory postsynaptic potential (EPSP) was normalized to the first one. Amplitudes and area were obtained using PClamp 10.2, and the statistical analyses using the GraphPad Prism 5.1 software. Differences were considered statistically significant at *p* < 0.05.

## Results

### Basal Synaptic Transmission in Schaffer Collaterals-CA1 in Young and Aged Female and Male Degus

#### Comparison by Sex

**FIGURE 1:**
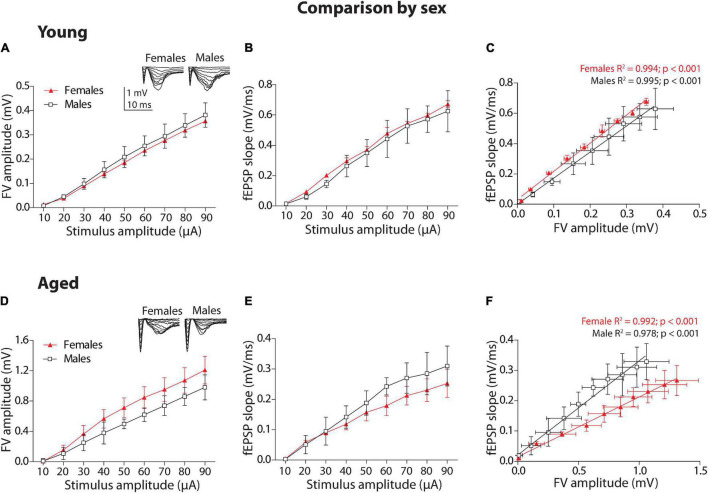
Sex comparison of the synaptic transmission basal parameters at Schaffer collateral-CA1 synapse of young and aged female and male degus. **(A,B)** Input–output relationship between different stimulus intensity (10–90 μA) and the fiber volley amplitude **(A)** or the field excitatory postsynaptic potential (fEPSP) slope **(B)** in young degus (**A,B**, 12–24 months old; *n* = 5 ♀, *n* = 5 ♂) and aged degus (**D,E**, 60–84 months old; *n* = 5 ♀, *n* = 5 ♂). The insets are representative recordings obtained by this protocol, showing the FV volley and fEPSPs evoked at increasing stimulation pulses (scale: 1 mV, 10 ms). **(C,F)** Correlation between fiber volley and fEPSP slope adjusted with a linear regression (values on the graph) in young **(C)** and aged **(F)** female and male degus. Two-way ANOVA followed by the Bonferroni *post hoc* test was used to analyze statistical differences in **(A,B,D,E)**; for **(C,F)**, we used an analysis of covariance (ANCOVA) test to analyze linear regression differences. Each symbol corresponds to the sex-age treatment group data represented as the mean ± SEM. At least three slices from each animal were considered replicates and averaged together. Black and red symbols represent the comparison by sex.

We performed electrophysiological protocols to determine whether there are differences in basal synaptic transmission between female and male of young degus (12–24 months old) and aged degus (60–84 months old). First, we compared the group of young animals by sex. To establish the relationship between the stimulus intensity and the extent of the evoked response, we applied increasing levels of current intensities to build an input–output (I-O) relationship between the fEPSP slope and FV amplitude (Figure 1A, inset). We plotted the FV amplitude versus stimuli amplitude (Figure 1A). The two-way ANOVA analyses showed a significant effect of stimulus intensity [*F*_(8_,_72)_ = 44.65, *p* < 0.001], but no effect of sex (*p* = 0.233) and no interaction between factors (*p* = 0.98). Then, we plotted the fEPSP slope against stimuli amplitude (Figure 1B). The same analysis showed a significant effect of stimulus intensity [*F*_(8_,_72)_ = 26.00, *p* < 0.001], but no effect of sex (*p* = 0.323), and no interaction between factors (*p* = 0.99). Then, in the next plot (Figure 1C), we correlated both parameters to determine the strength of synaptic transmission per activated axon during each stimulus intensity. We adjusted to linear regressions the correlations in female and male degus (*R*^2^ value on top of the graph); then, we compared both regressions using ANCOVA analysis as previously shown (Cisternas et al., 2019; Rivera et al., 2020; Viayna et al., 2021). Young female and male degus showed significantly different intercepts [*F*_(1_,_15)_ = 39.56, *p* < 0.001] but not different slopes (*p* = 0.07). This showed that female degus have more efficient synaptic transmission than male degus when young.

Second, we performed the same analysis for the group of aged animals. The plot of FV amplitude against stimulus amplitude (Figure 1D) and the two-way ANOVA analyses showed that there is a significant effect of stimulus intensity [*F*_(8_,_72)_ = 19.13, *p* < 0.001] and sex [*F*_(1_,_72)_ = 7.79, *p* < 0.01], but not on the interaction between these two factors (*p* = 0.97). Then, we plotted the fEPSP slope against stimulus intensity (Figure 1E). The two-way ANOVA analysis showed that stimulus intensity [*F*_(8_,_72)_ = 13.27, *p* < 0.001] but not sex (*p* = 0.076) or interaction (*p* = 0.97) are significant. We adjusted to linear regressions the correlation of fEPSP slopes and FV amplitudes between aged female and male degus (Figure 1F), and we compared them using ANCOVA analysis. In this case, aged female and male degus showed significantly different intercepts [*F*_(1_,_15)_ = 29.16, *p* < 0.001] and a different slope [*F*_(1_,_14)_ = 53.78, *p* < 0.001], indicating that more stimulus intensities in aged male degus displayed more efficient synaptic transmission than female degus.

#### Comparison by Age

**FIGURE 2:**
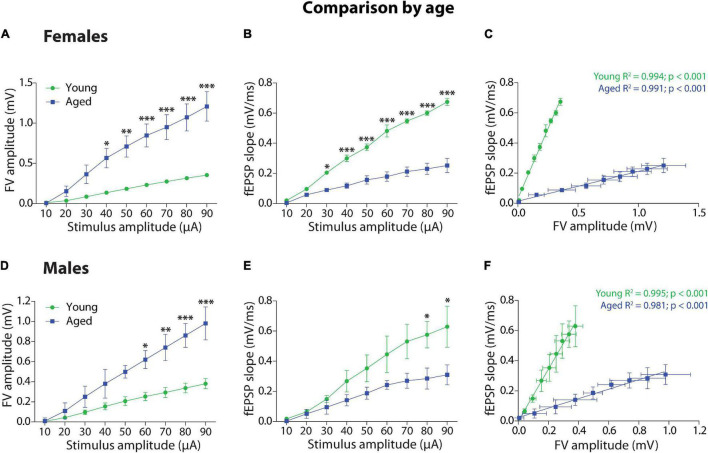
Age comparison of the synaptic transmission basal parameters at Schaffer collateral-CA1 synapse of young and aged female and male degus. **(A,B)** Input–output relationship between different stimulus intensity (10–90 μA) and the fiber volley amplitude **(A)** or the fEPSP slope **(B)** in young and aged female (**A,B**, 12–24 months old; *n* = 5 ♀, *n* = 5 ♂) and young and aged male degus (**D,E**, 60–84 months old; *n* = 5 ♀, *n* = 5 ♂). The insets are representative recordings obtained by this protocol, showing the FV volley and fEPSPs evoked at increasing stimulation pulses (scale: 0.5 mV, 10 ms). **(C,F)** Correlation between fiber volley and fEPSP slope adjusted with a linear regression (values on the graph) in young and aged female **(C)** and male **(F)** degus. Two-way ANOVA followed by the Bonferroni *post hoc* test was used to analyze statistical differences in **(A,B,D,E)**; for **(C,F)**, we used an ANCOVA test to analyze linear regression differences. Each symbol corresponds to data from the sex-age treatment group, represented as the mean ± SEM; *p < 0.05, ^**^p < 0.01, ^***^p < 0.001. Green and blue symbols correspond to the comparison by age.

Third, we compared by age the group of female degus (Figures 2A–C) and the group of male degus (Figures 2D–F). Within the female degus, the plot of FV amplitude against stimulus amplitude (Figure 2A) and the two-way ANOVA analyses showed that there is a significant effect on stimulus intensity [*F*_(8_,_72)_ = 16.63, *p* < 0.001], age [*F*_(1_,_72)_ = 115.93, *p* < 0.001], and on the interaction [*F*_(8_,_72)_ = 4.95, *p* < 0.001] between these two factors, indicating that the response to the stimulus depends on the age. The fEPSP slope versus stimulus intensity (Figure 2B) and the two-way ANOVA analysis shows that the stimulus intensity [*F*_(8_,_72)_ = 83.85, *p* < 0.001], the age [*F*_(1_,_72)_ = 382.58, *p* < 0.001], and the interaction [*F*_(8_,_72)_ = 18.35, *p* < 0.001] were also significant. We adjusted to linear regressions the correlation between fEPSP slopes and FV amplitudes between young and aged female degus (Figure 2C). The ANCOVA analysis showed that the intercepts were significantly different [*F*_(1_,_15)_ = 18.7, *p* < 0.001], but the slopes were not (*p* = 5.0), showing the efficiency of synaptic transmission in young versus aged female degus.

Within the groups of male degus, the plot of FV amplitude against stimulus intensity (Figure 2D) and the two-way ANOVA analyses showed that there is a significant effect of stimulus intensity [*F*_(8_,_72)_ = 16.17, *p* < 0.001], age [*F*_(1_,_72)_ = 59.16, *p* < 0.001], and interaction [*F*_(8_,_72)_ = 3.12, *p* < 0.01] between these two factors. Meanwhile, the fEPSP slope versus stimulus intensity (Figure 2E) and the two-way ANOVA analysis showed significant differences in the stimulus intensity [*F*_(8_,_72)_ = 11.74, *p* < 0.001], age [*F*_(1_,_72)_ = 24.25, *p* < 0.001], but not in the interaction (*p* = 0.199). The correlation between fEPSP slopes and FV amplitudes between young and aged male degus were adjusted to linear regressions (Figure 2F), and the ANCOVA analysis showed that the intercepts were significantly different [*F*_(1_,_15)_ = 20.09, *p* < 0.001] but the slopes were not (*p* = 5.0), showing the efficiency of synaptic transmission in young versus aged male degus.

To assess the relative importance of the stimulus amplitude on FV amplitude and fEPSP slopes, we performed an ordinary least squares (OLS) regression (Supplementary Figure 1). We showed how the stimulus amplitude has a higher effect across sex and age groups.

### Short-Term Plasticity in Aged and Male Degus

We used a pair-pulse protocol, also considered a short-term synaptic plasticity protocol, to determine the effect of presynaptic Ca^2 +^-dependent vesicle release on basal synaptic transmission (Zucker and Regehr, 2002; Jackman and Regehr, 2017). The PPF protocol measures the release probability of presynaptic terminals by applying two pulses separated by different inter-stimulus intervals. The ratio R2/R1 calculates the facilitation index, which is always above 1 in this synapse (R2/R1 > 1) (Schaffer collateral-CA1). The insets show examples of R1 and R2 pulses for each group at ISI = 20 ms (Example of traces at all ISIs in Supplementary Figure 2). We compared the PPF index between female and male degus and between young and aged female and male degus.

#### Comparison by Sex

**FIGURE 3:**
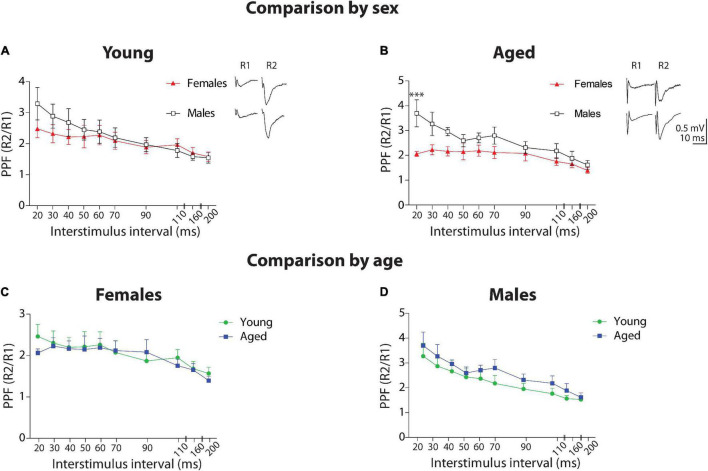
Short-term plasticity at Schaffer collateral-CA1 synapse of young and aged female and male degus. Comparison by sex. **(A–D)** Paired-pulse facilitation (PPF) obtained by plotting the R2/R1 ratio at different interstimulus intervals between 20 and 200 ms, in young **(A)** or aged **(B)** female and male degus (*n* = 5 ♀, *n* = 6 ♂). *Comparison by age*. PPF plot in young and aged female **(C)** or male **(D)** degus (*n* = 5 ♀, *n* = 6 ♂). The insets show representative traces of paired-pulse recordings at 20 ms ISI (scale: 0.5 mV, 10 ms). ***p < 0.001; black and red symbols represent the comparison by sex; green and blue symbols correspond to the comparison by age.

First, we compared the facilitation ratio and the time course between sexes (Figures 3A,B). In the group of young animals, the two-way ANOVA analysis showed a significant effect of stimulus interval [*F*_(9_,_90)_ = 3.79, *p* < 0.001], but no effect of sex (*p* = 0.147) and no interaction between factors (*p* = 0.85). This data indicates that Ca^2+^-related mechanisms involving synaptic vesicle release are not different between young female and male degus. Meanwhile, in the group of aged animals, the analysis found a significant effect of stimulus interval [*F*_(9_,_90)_ = 5.47, *p* < 0.001], sex [*F*_(1_,_90)_ = 28.21, *p* < 0.001], and no interaction between factors (*p* = 0.185), indicating that Ca^2+^-related mechanisms involving synaptic vesicle release are significantly different at shorter intervals. This data suggests a Ca^2+^-related mechanism in the presynaptic component of neurotransmitter release in aged male degus compared to female degus.

#### Comparison by Age

Next, we compared PPF by age (Figures 3C,D). In the group of female degus, the two-way ANOVA analysis showed a significant effect of stimulus interval [*F*_(9_,_90)_ = 2.7, *p* < 0.01] but not of age (*p* = 0.457) or interaction between the two factors (*p* = 0.988). When we compared between young and aged male degus, the analysis showed a significant effect of interstimulus interval [*F*_(9_,_90)_ = 6.54, *p* < 0.001], sex [*F*_(1_,_90)_ = 5.38, *p* < 0.05] but no significant effect of interaction (*p* = 0.96). Although the Bonferroni *posthoc* test did not show significance, the PPF in aged males appears to be enhanced compared to young males.

### Long-Term Synaptic Transmission in Young and Aged Female and Male Degus

#### Comparison by Sex

**FIGURE 4:**
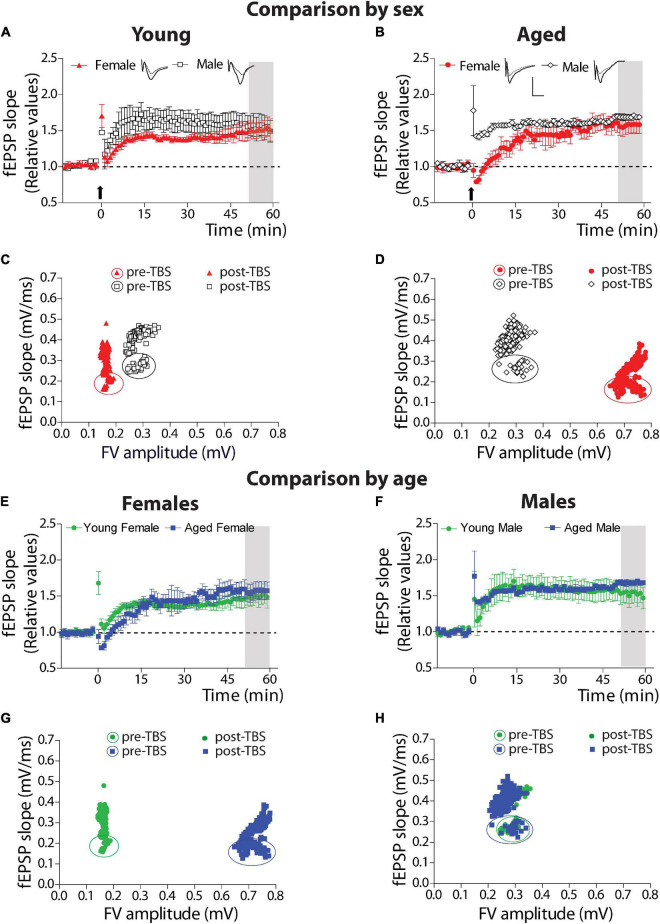
Long-term synaptic plasticity parameters in young and aged female and male degus. **(A–D)** Comparison by sex. **(A,B)** Stimulation with theta-burst stimulation (TBS) induces long-term synaptic plasticity (LTP) in the hippocampal Schaffer collateral-CA1 of young female and male degus **(A,C)** and aged female and male degus **(B,D)**. The arrow indicates the moment of the TBS application. The insets show representative evoked fEPSPs before and after LTP induction (scale: 0.5 mV, 10 ms). **(C,D)** Correlation between fiber volley amplitude and fEPSP slope of the pre-TBS and post-TBS components of the averaged data used to calculate LTP. Enclosed symbols indicate the pre-TBS points. *Comparison by age.* The plot compares young and aged female degus **(E,G)** and young and aged male degus **(F,H)**. The correlation between the pre-and post-TBS components is shown for female degus **(G)** and male degus **(H)**. Data represent the mean ± SEM (21 slices from *n* = 7 young ♀, 27 slices from *n* = 9 young ♂, 15 slices from *n* = 5 aged ♀, and 21 slices from *n* = 7 aged ♂ degus). One-way ANOVA followed by the Bonferroni *post hoc* test was used to analyze statistical differences at the last 10 min on each plot of LTP (gray area). We found the correlation between FV amplitude and fEPSP slope using Pearson’s correlation. Using Fisher ‘*r*’ to ‘*z*’ transformation, we used *z*-scores to compute the significance of the difference when we compare two correlation coefficients before TBS or after TBS. We found significant differences in all group comparisons (for details, see text).

We compared synaptic plasticity displayed by this hippocampal synapse of young female and male degus at different ages (Figure 4). We applied a protocol to study LTP. We plotted the fEPSP slopes generated after TBS (arrow), relative to the basal activity before TBS, in both young female and male groups (Figure 4A). As we observed, both young female and male degus showed a significant increase of plasticity over the basal level (Comparison of the last 10 min: female degus: 48.34 ± 12.22%; male degus: 53.96 ± 14.91%, above the baseline; Figure 4A) without differences between them (*p* = 0.34). To determine the synapse status before and after synaptic plasticity induction, we determined the efficacy of synaptic strength before and after LTP induction. We plot the correlation between the FV amplitudes and their corresponding fEPSP slopes and evaluate whether they differ between sexes (Figure 4C). Every point represents the averaged FV value, and the corresponding averaged slope of its evoked response from all the data is used to determine LTP. We built the plot of the data before (enclosed symbols) and after TBS (symbols) (Figure 4C). Interestingly, during basal stimulation, young female degus recruit fewer axons (FV amplitude) than male degus (female degus: 0.169 ± 0.003 mV vs. male degus: 0.239 ± 0.007 mV; *p* < 0.01), and they also generate lower evoked responses (fEPSPs slopes) than male degus (female degus: 0.223 ± 0.004 mV/ms vs. male degus: 0.279 ± 0.004 mV/ms; *p* < 0.05). During the post-TBS period (induction of LTP), we observed the same recruited axons in young female and male degus but with different evoked responses, as expected from the plasticity induction in the postsynaptic region (female degus: 0.333 ± 0.002 mV/ms vs. male degus: 0.427 ± 0.002 mV/ms; Figure 4C). By Fisher ‘*r*’ to ‘*z*’ transformation, we analyzed the correlation between variables, and we found significant differences between the group of female and male degus before and after TBS (*p* < 0.001). The normalized LTP responses were similar (Figure 4A) despite the differences.

We used the same protocols to study the synaptic plasticity of aged female and male degus. The protocol to generate LTP showed that both aged female and male degus could generate plasticity over basal level after TBS (Comparison of the last 10 min: female degus: 57.28 ± 12.06%; male degus: 67.37 ± 3.47%, above the baseline; Figure 4B) with no differences between them (*p* = 0.44). The correlation between the FV amplitudes with their corresponding fEPSP slopes determined the status of the synapse before and after to induce synaptic plasticity in aged animals (Figure 4D). During basal stimulation (enclosed symbols), aged female degus recruit twice the amount of axons (FV amplitude; female degus: 0.699 ± 0.005 mV vs. male degus: 0.389 ± 0.026 mV) but generate lower evoked responses (fEPSPs slopes) than male degus (female degus: 0.187 ± 0.002 mV/ms vs. male degus: 0.294 ± 0.011 mV/ms). By Fisher ‘*r*’ to ‘*z*’ transformation, we analyzed the correlation between variables, and we found significant differences between the group of female and male degus before and after TBS (*p* < 0.001). During the post-TBS period, aged female and male degus showed similarly recruited axons as basal conditions and different evoked responses expected from LTP generation in the postsynaptic region (female degus: 0.277 ± 0.002 mV/ms vs. male degus: 0.406 ± 0.003 mV/ms; Figure 4D). These data show that young female degus can evoke similar LTP by recruiting lower fibers than young male degus. However, aging needs twice more.

#### Comparison by Age

When we compared the effect of age within the group of female and male degus, we found no significant differences between the young and aged female degus (*p* = 0.166, Figure 4E) and between the young and aged male degus (*p* = 0.265, Figure 4F). The correlation between the FV amplitudes with their corresponding fEPSP slopes was also studied in young and aged female degus (Figure 4G) and male degus (Figure 4H). During basal stimulation (enclosed symbols), aged female degus have to recruit 4 times more axons than young female degus (young female degus: 0.169 ± 0.003 mV; aged female degus: 0.699 ± 0.005 mV) to generate similar evoked fEPSPs responses. On the other hand, young and aged male degus are close to being overlapped (young male degus: 0.239 ± 0.007 mV; aged male degus: 0.389 ± 0.026 mV). By Fisher ‘*r*’ to ‘*z*’ transformation, we analyzed the correlation between variables, and we found significant differences between the group of young and aged female degus (*p* < 0.001) but not between young and aged male degus (*p* = 0.471). This data strongly suggests that aged female degus, but not aged male degus, have less functional axons.

### Attenuation as a Form of Plasticity

We previously studied the presynaptic effect during basal transmission using the PPF protocol. However, together with the induction of plasticity, the TBS also induces the attenuation of the PPF response (Schulz et al., 1994; Wang and Kelly, 1997). This data indicated that synaptic changes during LTP involve a postsynaptic and a presynaptic component. To evaluate this, we plotted fEPSP of R1 and R2 pulses (%) and the PPF (R2-R1)/R1(%).

#### Comparison by Sex

**FIGURE 5:**
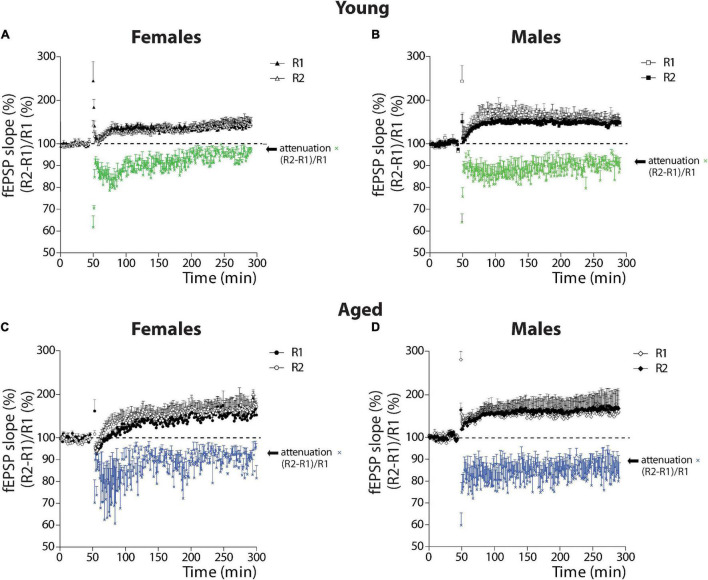
Analysis of attenuation as a form of plasticity in young and aged female and male degus. **(A–D)** Plots of the relative contribution of R1 and R2 evoked responses (%) (black and white symbols) and the calculation of the attenuation as (R2-R1)/R1 (%) (colored symbols) in young female degus **(A)**, young male degus **(B)**, aged female degus **(C)**, and aged male degus **(D)**. Data represent the mean ± SEM (21 slices from *n* = 7 young ♀, 27 slices from *n* = 9 young ♂, 15 slices from *n* = 5 aged ♀, and 21 slices from *n* = 7 aged ♂ degus). Two-way ANOVA was used to calculate the differences between young **(A,B)** or aged **(C,D)** female and male degus, including time-course and sex as factors; and between young and aged female degus **(A,C)** or male degus **(B,D)**, including time-course and age as factors. One-way ANOVA followed by Bonferroni *post hoc* test was used to analyze attenuation statistical differences at the last 10 min of LTP.

We first compared young female and male degus (Figures 5A,B). The two-way ANOVA showed that the attenuation of the PPF response in young female and male degus develop and differ significantly [sex: *F*_(1_,_1936)_ = 78.05, *p* < 0.001] at the beginning of the LTP induction [time: *F*_(241_,_1936)_ = 2.15, *p* < 0.001], with no interaction between factors (*p* = 0.95). Further analysis demonstrated that TBS induces the attenuation of PPF in both sexes, but in female degus, the attenuation of PPF is not sustained along with the development of LTP (Figure 5A). The last 10 min of attenuation analysis shows that young female degus are different from young males (*p* < 0.001; see Supplementary Tables 1,2). This data suggest that TBS modulates or exerts a differential regulation of a presynaptic mechanism depending on sex and age.

Like with young animals, we evaluated the attenuation of the PPF response in the aged animals (Figures 5C,D). The two-way ANOVA showed that the attenuation in aged female and male degus differ significantly (sex: *p* < 0.001) but not along with the development of LTP (*p* = 0.990), and with no interaction between factors (*p* = 0.98) (Figures 5C,D). The last 10 min of attenuation analysis shows that aged female degus are different from aged male degus (*p* < 0.001; see Supplementary Tables 1,2).

#### Comparison by Age

The comparison between the attenuation in young and aged female degus is significantly different [age: *F*_(1_,_1936)_ = 73.73, *p* < 0.001] like the time course [time: *F*_(241_,_1936)_ = 1.81, *p* < 0.001], and no interaction was found between these two factors (*p* = 0.95). The last 10 min analyzed with one-way ANOVA showed that young and aged females differed (*p* < 0.001). Meanwhile, significant differences were found between young and aged males [age: *F*_(1,1936)_ = 102.40, *p* < 0.001], but with no differences in the temporal course (*p* = 0.88) or in the interaction (*p* = 0.91). The last 10 min comparison between the young and aged males shows significant differences (*p* < 0.001). Altogether, these data indicate that along with the LTP development, the attenuation of PPF response depends on sex and age, and except in young female degus, is sustained along with the induction of LTP.

### Burst Analysis During Theta-Burst Stimulation Induction

#### Burst-First Pulse Comparison

**FIGURE 6:**
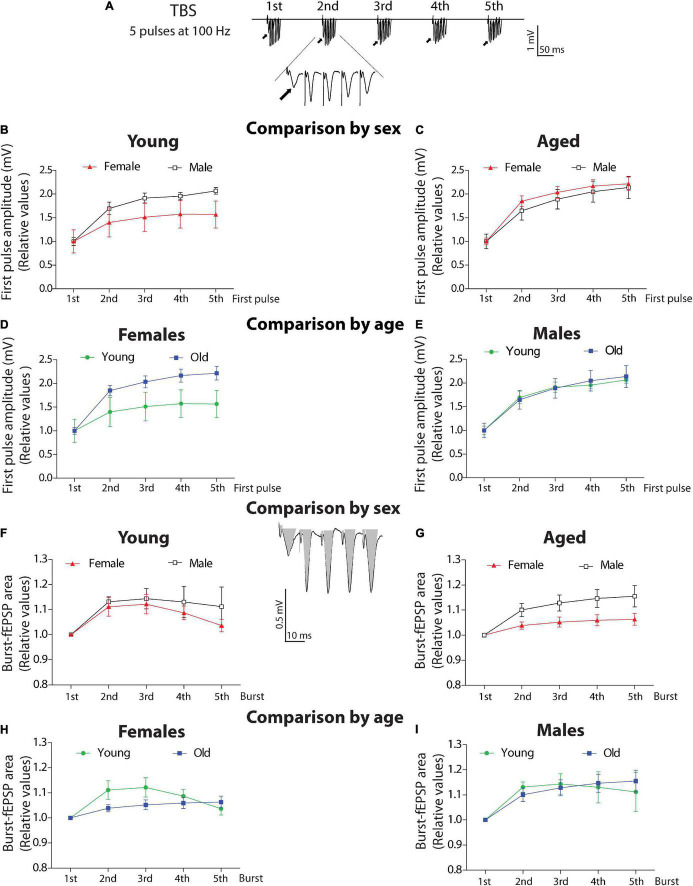
Analysis of burst activity in young and aged degus. **(A)** TBS scheme including five bursts (scale: 1 mV, 10 ms). **(B–E)** Analysis of the first pulse amplitude. Comparison by sex. The first amplitude of every burst was normalized and compared in young **(B)** or aged **(C)** female and male degus. *Comparison by age.* The first amplitude of every burst was normalized and compared in young and aged female **(D)** or male degus **(E)**. **(F–I)** Analysis of fEPSP area. *Comparison by sex.* Total evoked fEPSP area induced by burst was measured as shown in gray (scale: 0.5 mV, 10 ms). The 2nd, 3rd, 4th, and 5th burst areas were normalized to the area of the 1st burst, compared in young **(F)** or aged **(G)** female and male degus. *Comparison by age*. The fEPSP areas in young and aged female **(H)** or male degus **(I)**. Each symbol corresponds to data from the sex-age treatment group, represented as the mean ± SEM (21 slices from *n* = 7 young ♀, 27 slices from *n* = 9 young ♂, 15 slices from *n* = 5 aged ♀, and 21 slices from *n* = 7 aged ♂ degus). Two-way ANOVA was used to calculate the differences, including the bursts, sex, or age, as factors when corresponding.

To study the evoked responses induced during a train of high-frequency stimulation, we analyzed the evoked bursts during TBS application. We applied a TBS that generates burst stimulation that contains five pulses at 100 Hz (Figure 6A; examples of TBS analyzed for data see Supplementary Figure 3). In this analysis, we compared the amplitudes of all the first pulses of each burst (1st to 5th) along with the TBS and compared them between female and male degus at different ages (Figures 6B,C, burst inset, arrows). We normalized each amplitude to the amplitude of the first burst. The two-ANOVA analysis demonstrated that in the group of young animals, the amplitudes of the first pulses was significantly different [*F*(_4_,_55_) = 6.9, *p* < 0.001] like the effect of sex [*F*(_1_,_55_) = 7.71, *p* < 0.01], but with no interaction between these two factors (*p* = 0.691; Figure 6B). However, in the aged group, there is a significant effect in the amplitude between bursts [*F*(_4_,_55_) = 11.86, *p* < 0.001], with no effect of sex (*p* = 0.3862), and no interaction between factors (*p* = 0.989; Figure 6C).

Surprisingly, when we compared by age, aged female degus show higher amplitudes than young female degus (Figure 6D). The two-way ANOVA shows differences in amplitude in female degus at both ages [*F*_(1_,_40)_ = 10.13, *p* < 0.01] and differences along the bursts [*F*_(4_,_40)_ = 5.62, *p* < 0.01] with no significant interaction (*p* = 0.603). This can be explained by the number of axons recruited to compensate for the lower efficient transmission. In addition, no differences were detected between young and aged male degus (*p* = 0.847) or interaction (*p* = 0.989), but significative differences were found in the amplitude between bursts [*F*_(4_,_70)_ = 15.39, *p* < 0.001], showing that within the burst, male degus shows facilitation (Figure 6E).

#### Burst-Area Comparison

We also evaluated the total area of burst-fEPSP evoked for each burst stimulation (see inset, gray area), which is a measurement of excitation. We found no difference between young female and male degus (*p* = 0.286) – not in the area within bursts (*p* = 0.048) or the interaction between factors (*p* = 0.945; Figure 6F). The relationship became significant in the aged groups (Figure 6G), where aged male degus have a more evoked area [sex: *F*(_1_,_55_) = 11.23, *p* < 0.01], significant differences between bursts [*F*(_4_,_55_) = 4.36, *p* < 0.01], and no interaction between factors (*p* = 0.546). These data indicate that having more amplitude in the first pulse does not correlate with the strength of the total evoked response as these were lower in aged female degus than in male degus. It suggests that other compensatory mechanisms are involved in producing similar LTP.

The same parameters compared by age shows that between young and aged female degus, there are no differences (*p* = 0.069), but there are differences in the area within bursts [*F*(_4_,_55_) = 4.09, *p* < 0.01] and no interaction between factors (*p* = 0.194; Figure 6H). In the group of male degus, similar observations show no differences between young and aged (*p* = 0.919), but they do differ in their area during the TBS [*F*(_4_,_70_) = 4.17, *p* < 0.01]. No significant differences in the interaction between factors are observed (p = 0.914) (Figure 6I). This data suggests that male degus keep their high frequency evoked activity more stable throughout life.

See the Supplementary Figures for summary tables that contain all the statistical analysis reported here (Supplementary Tables 1, 2).

## Discussion

The present work studied sex and age-dependent differences in the electrophysiological parameters generated during basal synaptic activity and synaptic plasticity protocols. We used female and male degus at two different age ranges to assess that. We found that at young ages (12–24 months old), female and male degus showed similar characteristics, such as input–output responses, despite female degus being more efficient than male degus on basal synaptic communication. We found the opposite in aged animals (60–84 months old), where aged male degus showed better synaptic efficiency than female degus. The younger animals always display enhanced synaptic transmission compared to the aged animals in female or male degus. The measurement of short-term plasticity in paired-pulse facilitation showed that aged male degus have a better index than the same-aged female degus. In contrast, aged male degus are better than young male degus, suggesting a compensatory mechanism related to Ca^2+^-dependent vesicle release in presynaptic sites. On the other hand, the induction of long-term synaptic plasticity reveals no differences by sex in young or aged animals, reaching the same amount of LTP. However, the status of the synapse at the moment of TBS is significantly different in female and male degus of different ages. Young female degus recruit significantly lesser axons per evoked activity than young male degus. However, aged female degus have to use twice as many axons to reach the same amount of LTP as male degus, being significantly less effective in aged animals. Meanwhile, aged male degus display similar evoked potentials due to the same amount of axons recruited when young. Moreover, the amount of synaptic evoked potentials during burst activity is lower in aged female degus than male degus, showing that aged female degus are less able to sustain high frequencies. In general terms, the outcome of the synaptic plasticity is similar in both sexes but undoubtedly conveys other mechanisms to compensate for the response. Altogether, these data showed the impact of aging on some neuronal mechanisms and the strong effect of sex on the efficiency of synaptic transmission. To our knowledge, this is the first study reporting the interaction between two factors like sex and age in a mammalian model and represents the first approach to incorporate sex differences in studies involving aging progression.

*Octodon degus*, as a highly engaged social mammal, represent a great model to study sex-related physiological and neurological attributes, social-related cognitive status, and age-related aspects of all of them (Colonnello et al., 2011; Quispe et al., 2014; Bauer et al., 2019). Degus are socially plural breeder animals with community care of offspring. Nearly all (>95%) of the adult female degus in a group reproduce (Hayes et al., 2009; Ebensperger et al., 2011; Wey et al., 2013) and several group members share parenting (Silk, 2007). In addition, under laboratory conditions, female degus provide milk to their own and non-descendant offspring (Ebensperger et al., 2006; Jesseau et al., 2009). The occurrence of complex behaviors like those mentioned requires knowing the basis of neuronal communication, including the multiple factors that can modify it. We included sex as a factor to evaluate similarities or differences between female and male degus and aging due to the possibility that age could modify the behavioral outcome of degus. Moreover, following their long aging process in the controlled ambient of a laboratory gives us the confidence to assume that the changes of complex physiological processes during aging are due to the same conditions in all our animals.

In the present work, we demonstrated that aged female degus recruit fewer axons per stimulus than aged male degus, affecting how the brains of aged female degus can respond to stimuli. From our study, at least two questions arise. The first question is why aged female degus may have fewer axons or functional axons entrained in physiological functions? Furthermore, is this physiologically relevant considering that the output is not different from that in male degus? In previous work, we observed that in conditions of chronic isolation stress, male degus display more efficient basal synaptic transmission than female degus but are more susceptible to defects in synaptic plasticity due to stress (Rivera et al., 2020). Although the study was only in young animals, at least in terms of LTP, female degus can have additional mechanisms to overcome stressful conditions better than male degus, at least when young. In terms of social memory, female degus are more susceptible than male degus to the stressful experiences occurring early in life where isolation periods affect socialization and recognition memory in adult life (Rivera et al., 2021a). It means that female and male degus have different physiological mechanisms to solve complex experiences.

What aspects underlie the differential effect of age in female and male degus? Hormones shape our body and brain along with life (Seeman, 1997; Zárate et al., 2017; Bauer et al., 2019). Sex hormones, such as estradiol and progesterone, have receptors expressed in the entire brain, especially in regions relevant for emotions and memory, like the amygdala and hippocampus. Besides the one formed in ovaries, estrogens are also synthesized in the brain, and their receptors are present in spines, dendrites, and axons, suggesting that their effect is very local and specific (Markou et al., 2005; Hara et al., 2015; Zárate et al., 2017). Precisely, sex hormones can stimulate the spine density and the formation of synaptic structures, influencing the hippocampal function (Woolley and McEwen, 1993; Barth et al., 2015). The effect of estrogens in young animals involves spinogenesis and enhancement of synaptic connectivity, but with no changes in *N*-methyl-D-aspartate receptor (NMDAR)-mediated transmission. However, in the aged hippocampus, one of the effects of estrogen is to mobilize NMDA receptors from extrasynaptic sites to the synaptic space to restore the synapses “youth” but with no formation of new spines or synaptic structures (Adams et al., 2004). In the aged brain, this mobility of receptors counteracts the lower spine density and the detrimental number of axospinous synapses that estrogen cannot reverse with age (Adams et al., 2004).

The reduced estradiol levels could explain that our aged female degus show less functional axons. Changes in NMDAR mobility could explain, in part, why LTP maintains in aged female degus despite the reduction in the spine density at CA1 (Adams et al., 2001). The application of estradiol in brain slices of male degus and ovariectomized female rats increases the excitatory transmission and the generation of LTP independently of TBS (Woolley and McEwen, 1993; Kramár et al., 2013). On the other hand, estrogen activates the actin polymerization in spines during LTP (Shepherd, 2001; Norbury et al., 2003). Some of these modifications occur in minutes through the stimulation of the actin cytoskeleton, suggesting the existence of different pools of spines. Stable spines forming stable synaptic structures would develop during young ages when hormonal actions are constant and significant. However, other more dynamic spines form or deplete in days when hormonal levels decay (Woolley and McEwen, 1993; Bloss et al., 2011; Kramár et al., 2013; Hara et al., 2015). In our data, the remaining stable axospinous synapses at CA1 beyond hormonal decay in aged female degus could be the reason why LTP stays constant despite the reduction of other parameters. It remains to be elucidated whether the high-frequency burst activity during TBS stimulation activates the actin polymerization to sustain LTP in those remaining spines. The relationship of estradiol with all these synaptic parameters in our animal model still needs to be demonstrated.

The estradiol increases the excitatory synaptic responses enhancing release probability of individual vesicles and inducing multivesicular release (Smejkalova and Woolley, 2010). This fact can explain the more efficient synaptic transmission in young female degus, specifically in low probability release synapses like the CA3-CA1 synapse (Smejkalova and Woolley, 2010). In this synapse, most of the synaptic vesicles are released in a second pulse, induced by the accumulation of Ca^2+^ between two pulses. Because of this, we measured the PPF during basal transmission, which is similar in young female and male degus. However, in aged animals, the PPF is significantly higher in male degus than female degus. Whether this difference is an effect of estradiol, we do not know. From another point of view, the increment in excitatory transmission caused by estradiol reduces PPF (Smejkalova and Woolley, 2010), changing a low-probability synapse into a high-probability synapse. It suggests that estradiol has a significant presynaptic effect. In our data, this change can explain the diminishment of PPF during the progression of LTP (attenuation) in young female degus.

An underestimated analysis is the study of the attenuation magnitude, which measures the change in PPF during the development of LTP. Before the induction of LTP by the TBS, the PPF is a constant value. The TBS induced the postsynaptic potentiation and reduced the PPF ratio, causing attenuation (Wang and Kelly, 1996, 1997). If the TBS has a long-term effect over the PPF, the attenuation will stay constant along the time course of LTP. If the TBS effect over the PPF is not permanent, the attenuation will not last, and the PPF ratio would return to the control level. In our data, in young male degus and aged female and male degus, the attenuation persisted along with LTP recording, suggesting that their presynaptic region becomes strongly influenced during LTP. Instead, in young female degus, the attenuation was not permanent, suggesting that, in this group, the postsynaptic mechanisms to sustain LTP become more relevant. The postsynaptic Ca^2+^ and Ca^2+^-related signaling proteins (CaMKII/PKC or CaN) are fundamental for synaptic potentiation and the attenuation of PPF (Wang and Kelly, 1997). The activation of CaMKII/PKC would increase synaptic potentiation, while activation of CaN would increase facilitation (Wang and Kelly, 1997). A previous observation demonstrated that the hippocampus of male degus is the only region where CaMKII is affected by stress (Rivera et al., 2021a), suggesting the adaptability to change CaMKII levels. More studies are necessary to determine the relevance of these proteins in normal and pathological conditions in this animal model or their influence by hormones like estradiol. The relevance of attenuation leads to the protection of synapses during the massive stimulation caused by TBS, integrating only the input from this stimulus and displacing others (Wang and Kelly, 1996).

In stressful conditions, there is a reported reduction of spine density in aged animals and a lack of plasticity for remodeling, i.e., the formation of new spines (Bloss et al., 2011). During a stress paradigm, the hippocampus and the prefrontal cortex experiences loss of thin and immature spines in young and aged animals, with no changes in the more mature spines (Bloss et al., 2011). However, once the stressful condition ends, the remodeling experience-dependent formation of new spines resumes only in the young, not aged animals, suggesting loss of plasticity in the aged brain (Bloss et al., 2011). Young female degus experiencing different stress isolation periods reduced their basal synaptic transmission efficiency compared with their counterpart male degus. However, the long-term potentiation was preserved better in these female degus than male degus (Rivera et al., 2020). The lack of plasticity for brain remodeling in aged or stressed animals suggests the prevalence of stability over changes (Bloss et al., 2011). Is the aging brain privileging stability over plasticity? Can a young synapse be transformed into an age synapse by a stressful experience? These questions are still unknown, but recent data in degus provide some clues. Aged female degus with high performance in long-term memory tasks have poor abilities in short-term memory. The opposite occurs in young female degus, where animals with high values on short-term memory have poor performance in long-term memory tasks (Rivera et al., 2021b). This data shows that synaptic mechanisms that privilege the use of mature and stable synapsis are prevalent in a condition where short-term or dynamic use of the synaptic transmission is disadvantageous (i.e., during aging or stress).

The evidence exposed here suggests that physiological changes occurring in age and sex-dependent manner are modulating the basic parameters of synaptic transmission that underlie the more complex outcomes. When the aging process begins and the estrogens are still circulating, there is no formation of new spines. The plasticity is maintained by the relocation of NMDA from extrasynaptic sites into the synaptic space. When the aging process is more advanced, and the estrogens are absent, the neuronal physiology preserves the stability leaving aside plasticity. Like stressful conditions, a young brain looks more stable than plastic. These data suggest that the formation of new memories requires dynamic changes (i.e., spines formation). Because of that, short-term experiences are more susceptible to being affected in the aging process. Instead, stability is prevalent in advanced ages and so do long-term memories.

Physiological changes with age have been more attractive since the life expectance of the population increased (López-Otín et al., 2013; Jockwitz et al., 2021). Due to the growth of the older human population, recent investigations allocate their observations to determine the cognitive status of older people, correlating how they use the information to resolve a task (Jockwitz et al., 2021). However, numerous considerations should be included, i.e., previous experience and brain status during acquisition or retrieval. Studies about sex differences have had more resistance. Several studies use the same sex and generalize the conclusions to the whole species (Beery, 2018). Sex differences are found between female and male degus when using strategies to find an escape hole during memory acquisition (Popović et al., 2010). These differences are not observables during memory retrieval (Popović et al., 2010), suggesting the existence of different mechanisms involved in the acquisition and consolidation of memory associated with sex. Likewise, the long-term synaptic plasticity or LTP, which subserves relevant mechanisms for memory establishment and consolidation, evoked similar responses in female and male degus despite differences during basal transmission (Rivera et al., 2020). Physiological studies that compare men against women can generate controversy. For instance, studies on the effects of sex/age in perceptual thinking, decision-making, or behavioral changes generate stereotypes that can stigmatize women/men at different levels. While some of these studies did not find differences in everyday performed activities (Hirnstein et al., 2019), others found that women/men adopt different strategies to process cognitive tasks (Jockwitz et al., 2021). Indeed, during the aging process, women and men display notorious differences regarding susceptibility to some pathologies. Furthermore, recent evidence demonstrates that older women are at a higher risk for dementia than men. Indeed, The Alzheimer’s Association estimates that almost 2/3 of AD patients in the United States are women. Several aspects could explain this elevated incidence. Some studies show that the decay of estrogen during and after menopause uncouples the metabolic pathways that “feed” the brain and set the “pathway” to neurodegeneration (Scheyer et al., 2018). Women carrying the ApoE-4 allele have two times more chances to develop AD than a man with the same allele (Fleisher, 2005; Damoiseaux et al., 2012). However, Aβ deposition or tau neurofibrillary tangles show patterns that do not differ between the sexes (Ferretti et al., 2018). When considering risk factors linked to sex, aspects like comorbidities, hormonal-related disorders, and demographic and geographical data should be included. Women from low-income countries with low education and poor incomes have a higher incidence of dementia (Prince et al., 2012). On average, women live longer than men. However, this has recently been discarded as a relevant factor. The fact is that cognitive decline decays faster in women than in men (Ferretti et al., 2018), which shows that specific-risk factors for this pathology, remain to be elucidated.

*Octodon degus* is a species whose behavior shows striking resemblance with the natural development of AD. The differences between female and male degus appear to have physiological and neurological correlates that are interesting to follow. In this work, we performed extracellular electrophysiological recordings, which means that there are some specific questions that we cannot solve by this technique. The correlation between FV amplitude and the fEPSP slopes is a functional measurement and indicates when synaptic transmission is truncated or less functional. Using this technique, we demonstrated the effectiveness of natural compounds or drugs acting on the presynaptic compartment to improve its efficacy (Cisternas et al., 2019; Viayna et al., 2021), and in the case of degus, we found that these parameters change by stressful conditions (Rivera et al., 2020). The reason why, during the aging process of degus, the synaptic transmission is less efficient is unknown. It could be the number of functional axons available or a mechanism, but it is a matter for future studies to understand using intracellular and histological techniques. Our data help to elucidate the basis of these differences in basal status, and it would help to understand how they derive in neuropathological conditions to offer prevention or treatment.

## Data Availability Statement

The original contributions presented in the study are included in the article/Supplementary Material, further inquiries can be directed to the corresponding author/s.

## Ethics Statement

The animal study was reviewed and all procedures were approved by the Bioethical and Biosafety Committee of the Faculty of Biological Sciences of the Pontificia Universidad Católica de Chile (CBB-121-2013).

## Author Contributions

CO contributed to the conceptualization, formal analysis, writing of the original draft, reviewing, editing, and visualization. DR contributed to writing, reviewing, editing, funding acquisition, and visualization. TM, FB, and NI contributed to writing, reviewing, editing, supervision, and funding acquisition. All authors contributed to the article and approved the submitted version.

## Conflict of Interest

The authors declare that the research was conducted in the absence of any commercial or financial relationships that could be construed as a potential conflict of interest.

## Publisher’s Note

All claims expressed in this article are solely those of the authors and do not necessarily represent those of their affiliated organizations, or those of the publisher, the editors and the reviewers. Any product that may be evaluated in this article, or claim that may be made by its manufacturer, is not guaranteed or endorsed by the publisher.
