# Supplementary material for: Differential Role of Sex and Age in the Synaptic Transmission of Degus (Octodon degus)
Source: Front Integr Neurosci. 2022 Feb 28;16:799147. doi: 10.3389/fnint.2022.799147 (PMC8918727; doi:10.3389/fnint.2022.799147)
Supplement: Supplementary file 1 [file Data_Sheet_1.PDF]

Supplementary Figures

Differential role of sex and age in synaptic neurotransmission in degus (*Octodon degus*)

Carolina A. Oliva[1*], Daniela S. Rivera[2], Trinidad A. Mariqueo[3], Francisco Bozinovic[4], Nibaldo C. Inestrosa[1,5*]

[1]Center of Aging and Regeneration UC (CARE-UC), Departamento de Biología Celular y Molecular, Facultad de Ciencias Biológicas, Pontificia Universidad Católica de Chile, Santiago, Chile;
[2]GEMA Center for Genomics, Ecology & Environment, Facultad de Estudios Interdisciplinarios, Universidad Mayor, Santiago, Chile;
[3]Centro de Investigaciones Médicas, Laboratorio de Neurofarmacología, Escuela de Medicina, Universidad de Talca, Av. Lircay S/N, Talca, Chile.
[4]Center for Applied Ecology and Sustainability (CAPES), Departamento de Ecología, Facultad de Ciencias Biológicas, Pontificia Universidad Católica de Chile, Santiago, Chile;
[5]Centro de Excelencia en Biomedicina de Magallanes (CEBIMA), Universidad de Magallanes, Punta Arenas, Chile.

*Corresponding author: Carolina A. Oliva; carolinaolivagutierrez@gmail.com

Nibaldo C. Inestrosa; ninestrosa@bio.puc.cl

Running title: Establishing the basis for synaptic neurotransmission in degus

Supplementary Figure 1. We performed an ordinary least squares (OLS) regression to assess the relative importance of stimulus amplitude on FV amplitude and fEPSP slopes. By this test, we were able to show how stimulus amplitude has a higher effect across sex and age groups. In addition to assessing the significance of the effects, this analysis allows us to quantify the effect size using the R2 value. **A**, OLS regression analysis showed that the intensity of the stimulus was able to explain 75 and 81% of the total variation of the FV amplitude for young females and males. While 65 and 86% of the total variation were observed for old females and males. **B**, In comparison, the intensity of the stimulus was able to explain 93 and 85% of the total variation of the fEPSP slopes for females and young males. At the same time, 67 and 89% of the total variation were observed for old females and males.

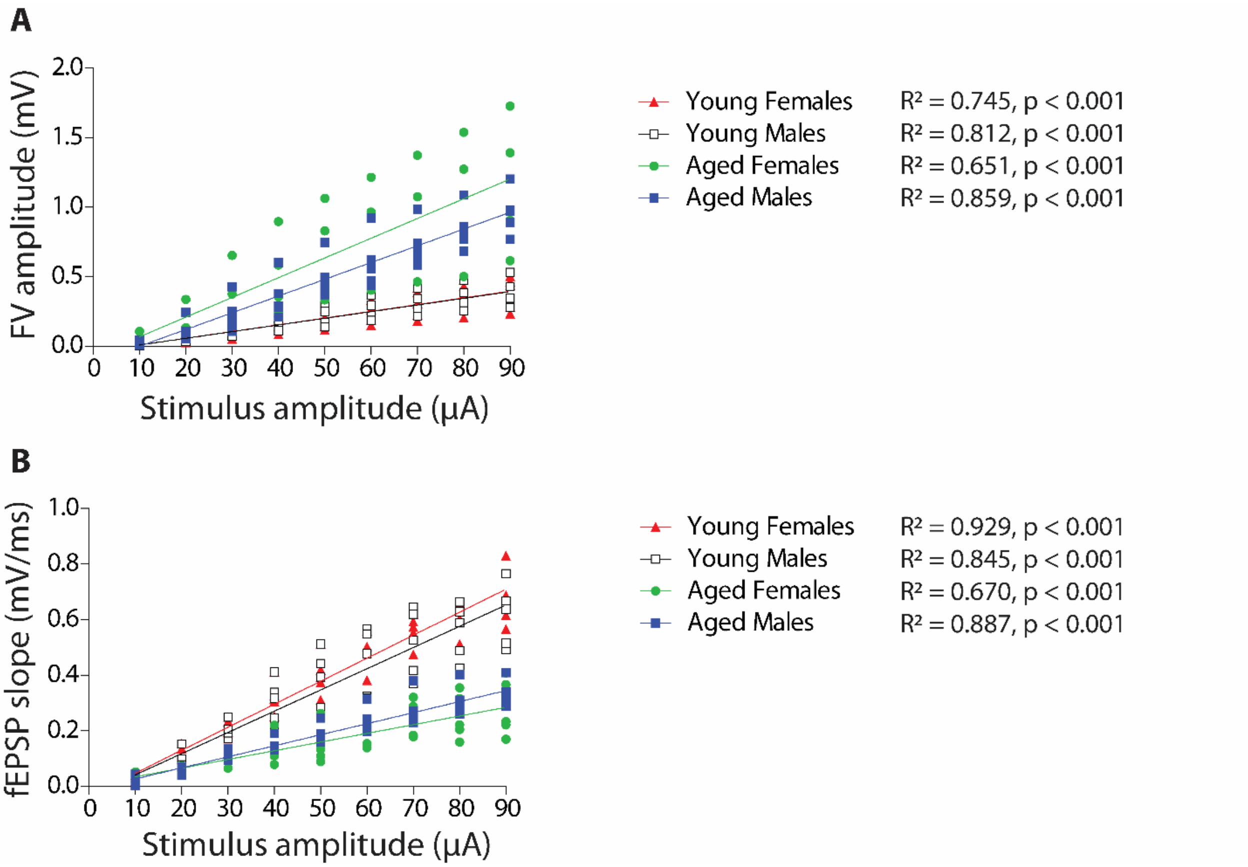

Supplementary Figure 2. Example of paired-pulse facilitation (PPF) experiment. It includes traces at all the interstimulus intervals (ISI) used in Figure 3.

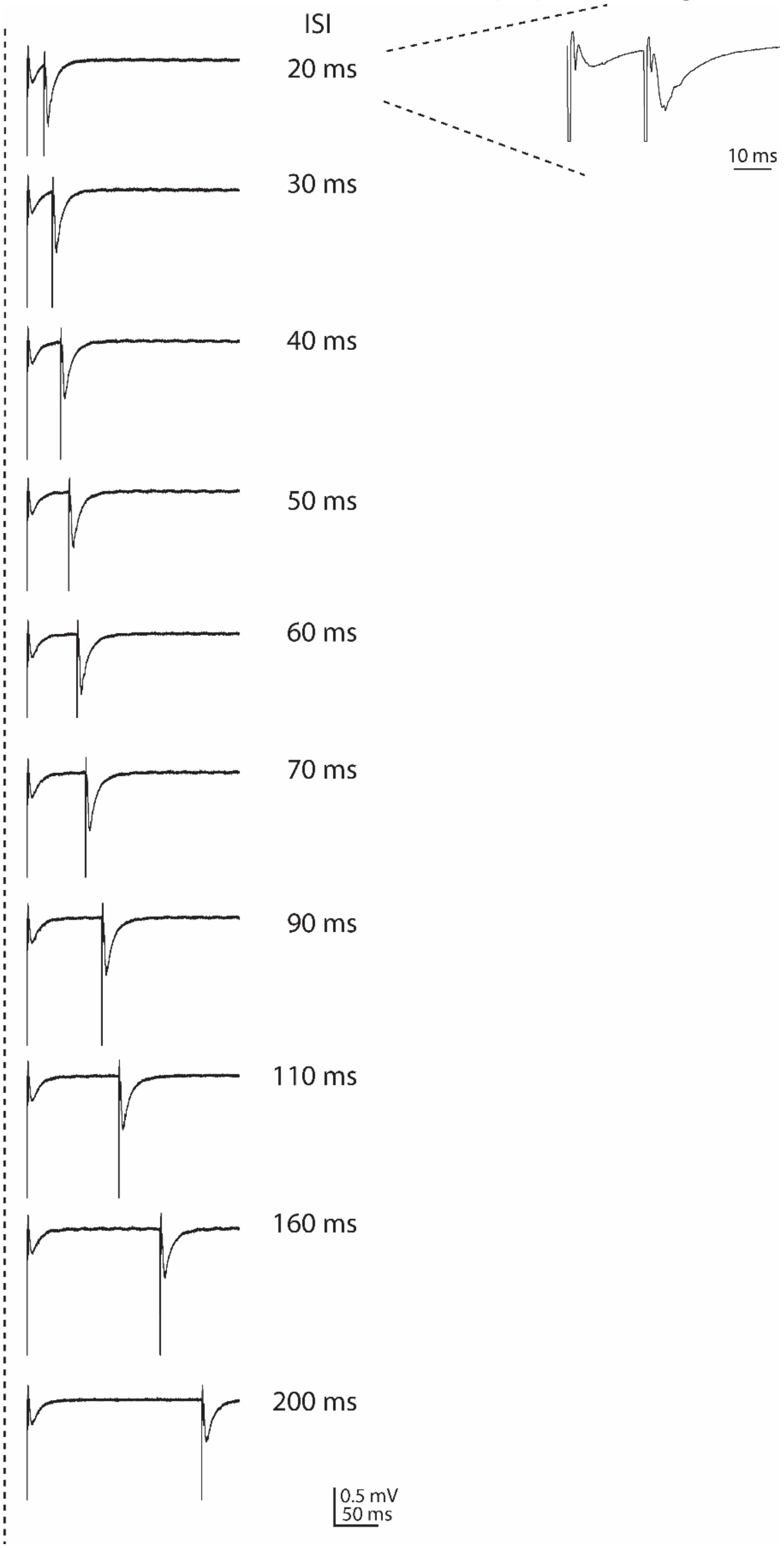

Supplementary Figure 3. Examples of TBS recording 'considered' and 'not considered' in the analysis. TBS consisted of five bursts; each burst formed by 5 pulses at 100 Hz. TBS was repeated every 20 s.

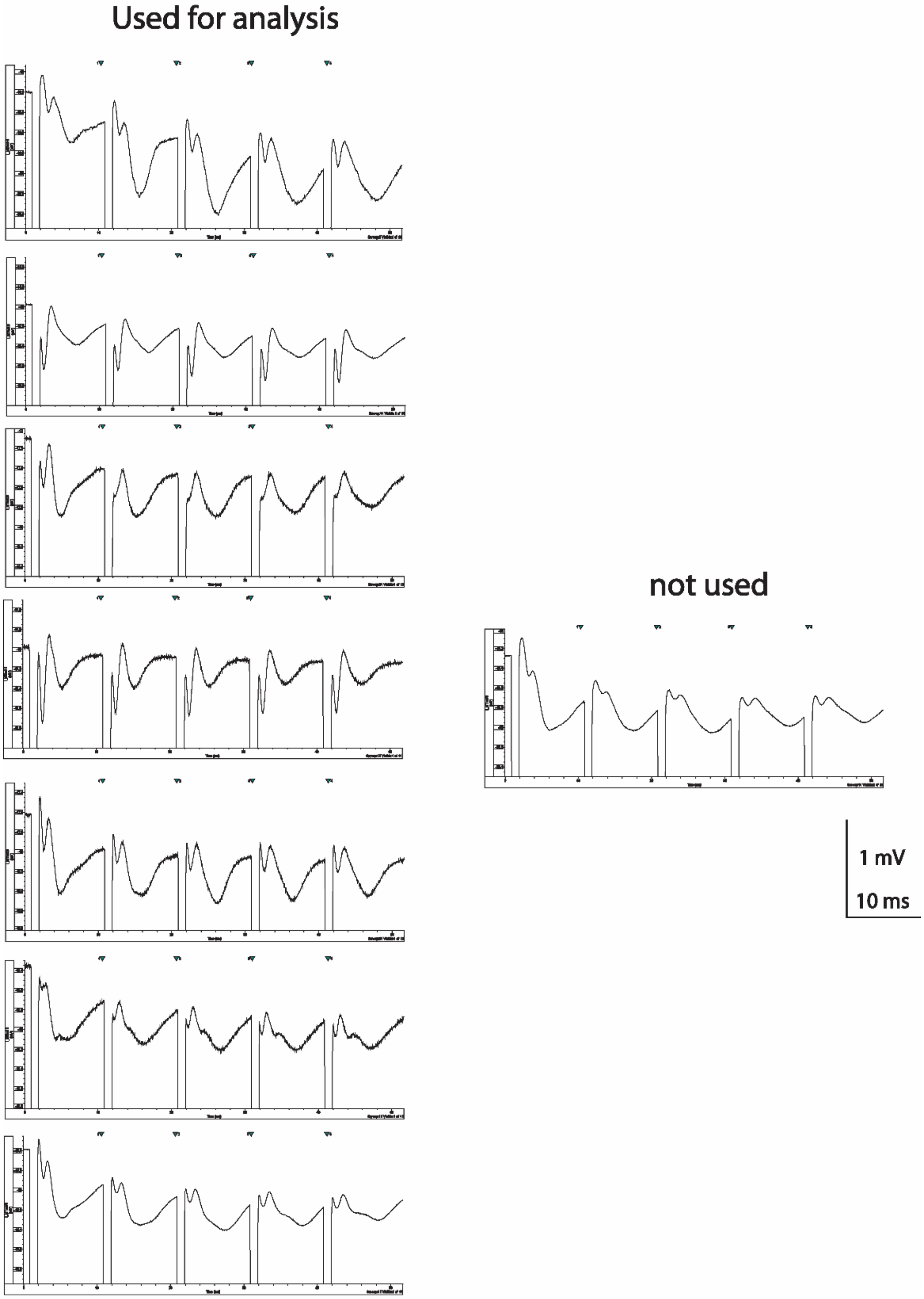

Table 1. Data includes the analysis using two-way ANOVA.

Input-Output			***Factor 1***		***Factor 2***	***interaction***
Comparison by sex			***stimulus intensity***		***sex***	***stimulus intensity x sex***
Young	I-O Fiber volley		$p < 0.001$		$p = 0.233$	$p = 0.98$
	I-O slope		$p < 0.001$		$p = 0.323$	$p = 0.99$
	Correlation	intercept	$p < 0.001$	slope	$p = 0.07$	
Aged	I-O Fiber volley		$p < 0.001$		$p < 0.01$	$p = 0.97$
	I-O slope		$p < 0.001$		$p = 0.076$	$p = 0.97$
	Correlation	intercept	$p < 0.001$	slope	$p < 0.001$	
Comparison by age			***stimulus intensity***		***age***	***stimulus intensity x age***
Females	I-O Fiber volley		$p < 0.001$		$p < 0.001$	$p < 0.001$
	I-O slope		$p < 0.001$		$p < 0.001$	$p < 0.001$
	Correlation	intercept	$p < 0.001$	slope	$p = 5.0$	
Males	I-O Fiber volley		$p < 0.001$		$p < 0.001$	$p < 0.01$
	I-O slope		$p < 0.001$		$p < 0.001$	$p = 0.199$
	Correlation	intercept	$p < 0.001$	slope	$p = 5.0$	

Paired-pulse facilitation (PPF)		***Factor 1***	***Factor 2***	***interaction***
Comparison by sex		***interstimulus interval (ISI)***	***sex***	***ISI x sex***
Young	PPF ratio	$p < 0.001$	$p = 0.147$	$p = 0.85$
Aged	PPF ratio	$p < 0.001$	$p < 0.001$	$p = 0.185$
Comparison by age		***interstimulus interval (ISI)***	***age***	***ISI x age***
Females	PPF ratio	$p < 0.01$	$p = 0.457$	$p = 0.988$
Males	PPF ratio	$p < 0.001$	$p < 0.05$	$p = 0.96$

Attenuation (R2-R1/R1)%				
Comparison by sex		***time***	***sex***	***time x sex***
Young	attenuation	$p < 0.001$	$p < 0.001$	$p = 0.95$
Aged	attenuation	$p = 0.99$	$p < 0.001$	$p = 0.98$
Comparison by age		***time***	***age***	***time x age***
Females	attenuation	$p < 0.001$	$p < 0.001$	$p = 0.95$
Males	attenuation	$p = 0.88$	$p < 0.001$	$p = 0.91$

TBS analysis (First pulse analysis)		***Factor 1***	***Factor 2***	***interaction***
Comparison by sex		***burst number***	***sex***	***burst number x sex***
Young	First pulse	$p < 0.001$	$p < 0.01$	$p = 0.691$
Aged	First pulse	$p < 0.001$	$p = 0.386$	$p = 0.989$
Comparison by age		***burst number***	***age***	***burst number x age***
Females	First pulse	$p < 0.01$	$p < 0.01$	$p = 0.603$
Males	First pulse	$p < 0.001$	$p = 0.847$	$p = 0.989$
TBS analysis (EPSP area)		***Factor 1***	***Factor 2***	***interaction***
Comparison by sex		***burst number***	***sex***	***burst number x sex***
Young	Area	$p = 0.048$	$p = 0.286$	$p = 0.945$
Aged	Area	$p < 0.01$	$p < 0.01$	$p = 0.546$
Comparison by age		***burst number***	***age***	***burst number x age***
Females	Area	$p < 0.01$	$p = 0.069$	$p = 0.194$
Males	Area	$p < 0.01$	$p = 0.919$	$p = 0.914$

Table 1. Data includes the analysis using two-way ANOVA. Summary of the statistical data obtained from the input-output, PPF, LTP, attenuation, and Burst-fEPSP experiments. Differences were considered statistically significant at $p < 0.05$.

Table 2. Data includes the analysis using on-way ANOVA.

Long-term potentiation (LTP)

Comparison by sex		***Females***	***Males***	***p***
Young	LTP	48.34 ± 12.22%	53.96 ± 14.91%	$p = 0.34$
	Basal FV (mV)	0.169 ± 0.003	0.239 ± 0.007	$p < 0.01$
	Basal fEPSP (mV/ms)	0.223 ± 0.004	0.279 ± 0.004	$p < 0.05$
	Post-TBS (mV/ms)	0.333 ± 0.002	0.427 ± 0.002	$p < 0.001$
Aged	LTP	57.28 ± 12.06%	67.37 ± 3.47%	$p = 0.44$
	Basal FV (mV)	0.699 ± 0.005	0.389 ± 0.026	$p < 0.01$
	Basal fEPSP (mV/ms)	0.187 ± 0.002	0.294 ± 0.011	$p < 0.05$
	Post-TBS (mV/ms)	0.277 ± 0.002	0.406 ± 0.003	$p < 0.001$
Comparison by age		***Young***	***Aged***	***p***
Females	LTP	48.34 ± 12.22%	57.28 ± 12.06%	$p = 0.166$
	Basal FV (mV)	0.169 ± 0.003	0.699 ± 0.005	$p < 0.001$
Males	LTP	53.96 ± 14.91%	67.37 ± 3.47%	$p = 0.265$
	Basal FV (mV)	0.239 ± 0.007	0.389 ± 0.026	$p = 0.471$

Attenuation (R2-R1/R1)%

Comparison of the last 10 min	***p***
Young females vs Aged females	***$p < 0.001$
Young females vs Young males	***$p < 0.001$
Young females vs Aged males	***$p < 0.001$
Young males vs Aged females	n.s.
Young males vs Aged males	***$p < 0.001$
Aged females vs Aged males	***$p < 0.001$

Table 2. Data includes the analysis using one-way ANOVA. Summary of the statistical data obtained from the LTP and attenuation experiments. Differences were considered statistically significant at $p < 0.05$
